# Bone Health in School Age Children: Effects of Nutritional Intake on Outcomes

**DOI:** 10.3389/fnut.2021.773425

**Published:** 2021-11-19

**Authors:** Steven A. Abrams

**Affiliations:** Department of Pediatrics, Dell Medical School at the University of Texas, Austin, TX, United States

**Keywords:** calcium, vitamin D, magnesium, phosphorus, rickets

## Abstract

The maximum rate of bone mass accumulation is during early adolescence. As such, a focus on optimizing mineral nutrition in school age children, defined here as approximately 5 to 15 years of age, is crucial to minimize the risk of bone loss that occurs later in life leading to osteoporosis and fractures. Optimizing bone mass in this age group requires attention to an overall healthy diet including adequate calcium, phosphorus, magnesium, and vitamin D. Special concerns may exist related to children who follow a restricted diet such as a vegan diet, those with intolerance or allergies to dairy, and those with chronic health conditions including young adolescents with eating disorders. Public policy messages should focus on positive aspects of bone health nutrition in this age group and avoid overly specific statements about the exact amounts of foods needed for healthy bones. In this regard, dietary recommendations for minerals vary between North America and Europe and these are higher than the values that may be necessary in other parts of the world. The management of many children with chronic illnesses includes the use of medications that may affect their bone mineral metabolism. Routine lab testing for bone mineral metabolism including the serum 25-hydroxyvitamin D level is not indicated, but is valuable for at-risk children, especially those with chronic illnesses.

## Nutritional Considerations in Healthy School-Age Children

### Calcium

Attention has primarily been paid to calcium intake, bioavailability, and turnover in this age group as a marker of bone nutritional adequacy. This is because calcium is the principal mineral contained in bone, and childhood, especially the early pubertal years, is the critical time period for calcification of the rapidly growing skeleton (1, 2). As such, calcium nutrition is critical to bone mineralization in children and public policy as well as dietary guidance has been focused on this critical age period. The evidence base behind the development of nutritional guidance for calcium has been derived from a variety of factors. These include extensive data from bone densitometry and related techniques demonstrating the usual rate of bone mineral accrual based on factors including sex and pubertal development (3). During growth changes over time in bone mineral content can be directly used to determine average calcium retention over a specified time interval, usually at least 6 months. This allows for a target for calcium retention to be established based on usual bone mineral accretion patterns.

Translating these data into dietary intake guidance has relied upon data evaluating calcium bioavailability, including both absorptive and excretory factors. These data, originally collected using traditional mass balance techniques, have been more recently enhanced with stable isotope labeling techniques. Stable isotopes of calcium are safe for children of all ages and they allow for an assessment of mineral metabolism with a high degree of reliability, without routinely collecting fecal samples. This makes such research much more acceptable to children and has allowed for numerous research studies assessing calcium absorption and bone calcium turnover in children. These studies have consistently shown a peak in calcium accretion and bone formation in early puberty, with a marked drop off by late puberty. This drop-off generally occurs at a younger age in girls than in boys. In girls, the peak bone mineral accrual is typically between 9 to 14 years of age with a delay of 1 to 2 years from this age common in boys (1, 4, 5). Overall, evidence suggests a fairly narrow window of 2 to 3 years during which calcium accretion to bone is maximized (4, 6).

The use of stable isotopic tracer techniques has further allowed for an evaluation of calcium absorption from dietary sources. Two critical examples of this are research showing that calcium absorption efficiency is very high from some vegetables, such as broccoli, but extremely low from spinach due to oxalate inhibition of absorption. Other dietary factors may affect calcium bioavailability including inulin-type prebiotics which enhance calcium absorption likely due to colonic scavenging of unabsorbed calcium from the small intestine (7, 8).

Dietary survey data has been critical in assessing usual dietary calcium intake but is often difficult to interpret due to inaccuracy of recall data used in most surveys. Overuse of cut-off values, especially the recommended daily allowance (RDA) and statements about a “calcium crisis” often associated with dietary usual only slightly below the RDA have not readily been borne out by short and long-term outcome data. Similarly, large amounts of calcium supplementation trial data indicate a short-term benefit to supplementation on bone mineral content, but few data consistently show a long-term or large-scale effect, and some controlled trials show no long-term benefit of calcium supplementation to levels above recommended dietary allowances (9).

This has led to questions about the utility of routinely supplementing children with calcium compared to focusing on adequate naturally occurring dietary calcium (10). Overall, the data are most consistent with a benefit to a diet in school-age children in which the calcium source is principally dairy or calcium-fortified dairy substitutes, and vegetables, and with the use of some fortified foods such as orange juice and cereals (11). There appears to be a synergistic effect of exercise and adequate calcium nutrition in adolescents (12) consistent with broad recommendations for exercise in this age group.

Most available data have been based on diets commonly consumed in industrialized countries, especially North America and Europe. Data from Asia and from Africa indicate that school age children have usual diets with calcium intakes that are often far below those recommended by either North American or European groups. Less outcome data is available for these populations, but in school age children, intakes of less than half of the US average often are frequently associated with little or no evidence of harmful effect (13). Whether this is due to other components of the diet, genetic variability, or later catch-up is not clear.

Despite limited long-term data, it is likely that substantial deficits in calcium and bone mineral intake do harm peak bone mass and thus increase the risks related to long-term bone loss. My research group evaluated whether adaptation would occur in diets commonly seen in the United States to very low calcium intake and found only partial adaptation with net calcium retention falling well below levels on higher intake (14). This follows earlier work looking at population data suggesting that regions of a country with low calcium intakes had lower ultimate bone mass (15).

Nutritional rickets, although rare after early childhood, may often occur in school age children associated with a combination of low calcium and vitamin D intake. Substantial adaptation to low calcium intake occurs with increased calcium absorption in the presence of adequate vitamin D. However, very low calcium intakes are not associated with an adequately positive calcium balance during puberty leading to long-term concerns for bone health even in the absence of radiologically apparent osteopenia or rickets (16, 17). Taken together, these data support global targets for calcium intake which may differ based on region but indicate that very low intakes should be avoided regardless of location and race. What exactly constitutes a very low intake risking rickets or fractures during childhood is uncertain, but populations with calcium intakes <300 to 400 mg per day seem to be at greatest risk (18). Whether this translates into a need for global fortification or supplementation strategies targeting very low intake populations is unclear at the present time. Such programs face obstacles related to costs and acceptability of fortification and supplementation strategies.

Implementation of strategies to increase calcium intake in school age children need to recognize the broad range of foods that can be used to provide calcium and the opportunity to include calcium containing foods in recipes used in meal preparation. Although cow milk remains a primary calcium source, there has been a decrease in cow milk intake among children over the past 40 or more years that continues to the present time (19). Cow milk has been replaced in the diets of many school children by non-calcium containing soda, juices, and flavored waters. Dietary guidance needs to recognize this change and consider counseling specific to the use of calcium fortified non-dairy products (e.g., orange juice, soy, and vegetable milk-type products) and other dairy products including yogurt that are increasing popular with this age group.

Taken together, these data indicate the importance of calcium nutriture in school age children, especially during peak bone growth of puberty, typically about 9 to 14 years of age. However, they also indicate caution in overinterpreting intake data both due to weakness in the available data, lack of data on populations with lower usual intake and relatively little long-term data clearly demonstrating benefits to high intakes in all populations.

### Vitamin D

Adequate vitamin D is necessary for the active, transcellular absorption of calcium. Adequacy of vitamin D status is usually assessed by measuring the serum 25-hydroxyvitamin D concentration (25-OHD) although the principal physiologically active form is the 1, 25 dihydroxyvitamin D which is produced in the kidneys. Although controversy exists about identifying values of serum 25-OHD that are considered adequate or optimal for both calcium absorption and other biological functions, most authorities, including the Institute of Medicine, use a value of 50 nmol/L as a target serum concentration as related to bone health (1, 20). Serum 25-OHD concentrations using higher target values of 80 or more nmol/L are not associated with meaningful increases in calcium absorption fraction and are difficult to achieve without the use of supplements (21, 22). Although values of 25-OHD below 50 nmol/L are common in school age children, there is relatively little evidence for a substantial harm to bone health in most children who are otherwise healthy to levels from 30 to 50 nmol/L (23). Greater concern exists for vitamin D insufficiency in children with chronic illnesses affecting either vitamin D absorption, metabolism, or calcium intake and utilization (24).

The Institute of Medicine established an estimated average requirement (EAR) in this age group of 400 IU/day (10 micrograms/day) and a recommended dietary allowance (RDA) of 600 IU/day. Intakes of 600 IU/day are difficult to achieve through diet alone in children unless they have a large dairy intake (1). Most dietary vitamin D in the United States is derived from fortified dairy products, especially milk which is almost universally fortified with vitamin D with only a very few organic dairies and “raw” milk farms not fortifying milk with vitamin D. However, decreased milk drinking over recent decades among school age children and adolescents has decreased that source of both calcium and vitamin D intake. Other commonly vitamin D fortified foods now include orange juice, cereals, flour (including bread), and mushrooms but total intakes remain below recommendations. The Dietary Guidelines for Americans 2020 recognized vitamin D intake as a nutrient of concern for low intake (25).

Whether school children should routinely take a vitamin D supplement, however, is controversial. The use of multivitamin and mineral supplement pills has not been shown to have clearly identified health outcome benefits for healthy children and adolescents who are not severely deficient in vitamin D or have abnormalities related to mineral metabolism (23). Nor is there evidence to recommend routine testing of serum 25-OHD in healthy children and it is not part of recommendations related to bone health from the American Academy of Pediatrics (2). Consideration of testing and supplementation would be stronger for those with recurrent fractures, those with chronic illnesses and those (see below) who avoid common sources of vitamin D in the diet such as dairy products.

The role of natural sunshine exposure in vitamin D status in school children is highly variable based on factors including season, latitude, use of sunscreens, skin characteristics and skin covering. As such, it is generally not assumed that natural sunlight exposure is adequate for any age group, including school age children (1). Nonetheless, children in the US during the summer, especially those who are commonly outdoors, generally show relatively higher levels of 25-OHD regardless of diet. Factors affecting serum 25-OHD levels in children include obesity, season of measurement and ethnicity/race although the effects of these on actual calcium absorption is less certain (26). Despite lower levels of 25-OHD for example, African American children and adolescents tend to absorb calcium at a higher efficiency than Caucasian children (27). Differences in renal handling of calcium may also occur between racial and ethnic groups affecting dietary requirements for both calcium and vitamin D (28).

### Other Key Dietary Factors

The roles of other mineral, especially phosphorus and magnesium, are often inadequately appreciated in maintaining adequate health. In school age children, a low phosphorus intake is uncommon except on a highly restricted diet with minimal dairy or meat. However, magnesium intakes can be below optimal for bone health on many typical diets. This is of concern as magnesium may be at least as critical as calcium intake for maximizing bone health in children (29). Concern has been expressed about the effects of excess phosphorus intake on bone mineralization, especially as might occur with high intakes of some soda beverages (30). Although some data suggest a negative effect of soda intake on bone mineralization, it is likely this effect is small (31). Much of the effect may be related more to displacement of healthy beverages rather than a direct effect of the soda. Soda intake should be limited in school age children as it has no health advantages and displacement of healthy beverages including dairy may be substantial with excess soda intake. However, a very limited amount of soda intake is unlikely to substantially harm bone development in children and caution should be used to not overstate the effects.

### Genetic Regulatory Factors Affecting Bone Health

Genes regulating vitamin D also play a role in calcium metabolism and bone health. Most of the attention has been paid to genes associated with the vitamin D receptor (VDR). Specific allelic variations of the VDR gene have found associations with calcium absorption, bone turnover and, in adults, osteoporosis (32–35). Further genetic analysis to understand variations in specific gene polymorphisms and calcium and bone metabolism are needed however, and the current studies are not adequate to provide any specific interventions for at-risk populations or indicate the need for broad genetic screening although individual case reports linking specific genetic polymorphisms and low bone mass and fractures exist (36).

## Nutritional Limitations

### Vegan or Other Restrictive Diets

It is increasingly common for school children to follow vegetarian diets. This can be either due to this being a family dietary pattern that they are part of, or as a decision they have made on their own. As with adults, the reasons for this decision are highly variable. However, it is likely the case that an increasing number of young adolescents are making this choice due to environmental sustainability as well as health related concerns (37). Some may be following strict limitations on animal products whereas for many others, both individuals and families, it may be a decision to decrease animal-based products in favor of plant products rather than a complete elimination of animal products.

Many schoolchildren who choose a vegetarian diet will continue to consume dairy products. In this population, most nutrients relative to bone health will be adequately consumed, especially calcium, and little specific additional guidance is needed.

Among those who avoid dairy, whether it is part of a strict vegan diet or more general dairy avoidance may face a substantial decrease in calcium intake as well as lower intake of vitamin D, magnesium, and phosphorus (38). Alternative sources include both soy milk and other plant beverages including those based on almonds. In this case, especially with the non-soy alternatives, a key factor is whether the product is fortified with calcium and vitamin D. This is highly variable among products and school children may not be experienced at reading a food label to identify which products are fortified. Education for the entire family is needed to read the food label and choose beverages that are calcium and vitamin D fortified when feasible.

Concern has been expressed that soymilk products have lower calcium bioavailability than dairy products. Data regarding this are mixed and although there may be a lower bioavailability of the calcium, it is not likely to be a major factor in overall calcium nutrition (39). The key to successfully following a dairy-free diet is to ensure that families are aware of how to interpret the food label and to target an adequate mineral intake, especially for calcium.

There is relatively little data regarding the bone outcomes of vegetarian or vegan diets. One recent study from Poland found lower bone mineral content in those who followed a vegan diet of about 3–6% (40). The clinical consequence of this difference is uncertain but may reflect the dietary issues mentioned above.

### Cow Milk Protein or Lactose Intolerance

Intolerance to lactose by school children can lead to lower intake of minerals, especially calcium. Although low lactose milk and other dairy products as well as enzyme pills which increase lactase are readily available, many do not use these products but instead decrease their dairy intake. Although true cow milk protein allergy is relatively uncommon, it also can have a substantial effect on dairy and therefore bone mineral intake. Education related to lactose intolerance and alternative products is critical to enhance bone health in these at-risk groups and evaluation of such children who present with clinical evidence of bone loss should be done (41).

### Eating Disorders and Obesity

Poor nutritional intake including a decrease in bone mineralization can commonly be seen in young adolescents with eating disorders, primarily anorexia nervosa. Often seen as part of the “athletic triad”, decreased bone mineralization can be associated with fracture which can be the presenting aspect of the eating disorders (2, 42). A full evaluation of young athletes and others who present with fractures should be done to assess for this possibility. Most concern about low peak bone mineral relates to the risks associated with bone loss in later life, but this population of young adolescents can present with serious functional impairment at the time of the decreased intake and eating disorder. Of note is that the bone loss physiology may include direct effects on calcium bioavailability and turnover related to the steroid hormone changes associated with eating disorders (43). This effect may also be related to delayed puberty in some girls with anorexia nervosa (44).

The effects of obesity in children on bone mineral status has been evaluated in several studies reviewed recently (45, 46). Bone mineral content and density are often relatively high in children with obesity, but fracture rates may be elevated suggesting the possibility of increased bone fragility. Considerable additional data are needed related to these relationships, but there are no data suggesting a benefit from higher intakes than usual of bone minerals in this population.

### Other Circumstances

Several recent studies have considered the effects of therapies used for transgender adolescents on bone mass. Lower intake of calcium and decreased physical activity may also be involved in leading to lower bone mineral density (47, 48). Physician awareness of this issue and consideration of full evaluation of these youths to include bone mineral assessment is necessary.

An increasing number of school age children have a chronic health condition that may affect bone health. These include liver and renal disease, epilepsy and its treatment, childhood cancer survivors and many others. Among those of special concern regarding bone health and nutrition are cystic fibrosis, inflammatory bowel disease, and intestinal failure due to “short gut” or other similar conditions. Genetic conditions, including Trisomy 21 and inborn errors of metabolism may also affect bone health. Rarer conditions include osteogenesis imperfecta, various endocrinopathies of the vitamin D and parathyroid systems, and progeria syndrome are associated with abnormalities of bone affecting the risks of fractures and/or rickets.

For most conditions not specifically associated with malabsorption of calcium, usual recommended amounts of calcium and vitamin D are recommended. Attempts to provide very high intakes of either of these is likely only to be successful in enhancing bone health when the underlying physiology suggests a utility to high intakes such as vitamin D-resistant rickets. Conditions related to nutrient malabsorption may benefit from increased calcium intake, but this can also cause digestive problems and may not be effective depending on the severity of the malabsorption. Considerable attention has been paid to dietary and supplement recommendations for children with cystic fibrosis. Although high dose vitamin D is often needed to achieve targeted 25-OHD level >75–80 nmol/L (49), specific benefits of this approach are less certain and require further evaluation (50).

Medication use can significantly affect bone health in school age children. Oral steroids severely affect both calcium absorption and bone turnover. Available bone mineral density data suggest the potential for an impact of inhaled steroids on both height and bone mineralization. However, data in children are mixed and the effect may be small if present (51, 52). Also of concern are some anti-epileptic medications, although the practical consequence of more commonly used ones in school children (e.g., levetiracetam) is likely minimal (53, 54). Immunosuppressive medications used in the treatment of auto-immune disorders may affect bone health and monitoring may be needed for these children. In general, for most children, medication use will be a small factor in bone health but need to be considered in some cases (55).

An uncommon condition that can affect bone health in children is “idiopathic juvenile osteoporosis.” The average age of onset is 7 years of age and can present with pain, difficulty walking or repeated fractures. Bone densitometry evaluation will show low bone mass and is more sensitive than routine X-rays. No therapy is routinely needed and referral for severe cases for possible use of bisphosphonates is needed. Most recover completely (56).

## Global Differences and Harmonization of Reference Intake Values

Reference vitamin and mineral intake values vary globally but two currently widely used ones are from the Institute of Medicine (IOM) covering the United States and Canada and the European Food Safety Authority (EFSA) (1, 57–61). There are some differences between these values and recent consideration of harmonizing the values (Table 1). It should be noted that there are fewer data with recommendations for low- and middle- income countries, but due to other factors in the diet, actual calcium needs may vary considerably from those of North America and Europe.

**Table 1:** Dietary reference values from the Institute of Medicine for the US and Canada (RDA, EAR and UL) and from the European Food Safety Authority (EFSA) (PRI and AI)^#^.

**Life-stage group**	**Calcium (mg/d)**				**Vitamin D (IU/day)**				**Magnesium (mg/d)**				**Phosphorus (mg/d)**			
**All children**	**RDA**	PRI (EFSA)^*^	**EAR**	**UL**	**RDA**	**AI (EFSA)**	**EAR**	**UL**	**RDA (diet)**	AI (EFSA)^**^	**EAR (diet)**	**UL (supplement)**	**RDA**	AI (EFSA)^*^	**EAR**	**UL**
4–8 y	1,000	800	800	2,500	600	600	400	3,000	130	230	110	110	500	440	405	3,000
**Males**																
9–13 y	1,300	1,150	1,100	3,000	600	600	400	4000	240	300	200	350	1,250	640	1,055	4,000
14–18 y	1,300	1,150	1,100	3,000	600	600	400	4000	410	300	340	350	1,250	640	1,055	4,000
**Females**																
9–13 y	1,300	1,150	1,100	3,000	600	600	400	4000	240	300	200	350	1,250	640	1,055	4,000
14–18 y	1,300	1,150	1,100	3,000	600	600	400	4000	360	300	300	350	1,250	640	1,055	4,000

# *RDA, Recommended Dietary Allowance; PRI, Population Reference Intake; EAR, Estimated Average Requirement; UL, Upper Limit; AI, Adequate Intake*.

* *Ages for PRI and AI are 4–10 instead of 4–8 and 11–17 instead of 9–13 and 14–18*.

** *Ages for AI for magnesium are 3–9 and 10–17*.

In comparing the EFSA values with those of the IOM, EFSA has chosen to use an adequate intake rather than an estimated average requirement (EAR) or RDA for magnesium, phosphorus, and vitamin D. They have provided a PRI (similar to an RDA) for calcium. Values chosen are generally slightly lower for the bone minerals from EFSA than from the Dietary Reference Intake (DRI) values from the US and Canada. Except for phosphorus however, where the EFSA derived adequate intake (AI) is much lower than the RDA or the EAR, these differences are relatively small and would not substantively affect meal planning. The lower phosphorus value would affect diets in the United States where the intake of phosphorus containing soda, meat and other sources make average intakes relatively high compared to the European standard.

Efforts to harmonize these values have been recently done based on a framework of careful evaluation of the basis for the values and consideration of physiological consequences of intake variations. Proposed values have been provided. Of note is that one proposal has suggested using IOM values for magnesium and phosphorus and EFSA values for calcium although this proposal has focused on the EAR equivalent values rather than the RDA values (62, 63).

There are relatively few specific guidelines from Asian countries for calcium and vitamin D intake, but these are generally similar to those from the United States and Canada and Europe. Guidelines from India tend toward the lower side of these recommendations consistent with generally lower intake of calcium in the diet (64).

Overall, further research is needed to understand physiological variability in children of all ages to develop ideal dietary recommendations. However, this ongoing process should not preclude an understanding of messaging to the public regarding the value of these key nutrients or be considered as representing a lack of value of a diet replete with bone minerals during growth.

## Recommendations for Research

There are several key gaps in knowledge which should be addressed through further research. One of these is a better understanding of the developmental basis, including genetic factors, involved in calcium absorption. There is little basis for understanding the precise gastroenterological changes which lead newborns to utilize vitamin D-dependent mechanisms for calcium absorption during the first months of life or after preterm delivery.

On a global basis, there is still inadequate research into the amount of bone minerals needed in the diet of populations who habitually have very low intakes, especially those in parts of Asia and Africa. Despite intakes that are generally far below amounts in the United States and Europe, as noted in this review, bone disease including osteoporosis are relatively uncommon in these populations and guidelines for calcium intake may inadequately reflect the needs of these populations.

Finally, the interaction of magnesium and trace minerals with the primary bone minerals has been minimally studied in children. Guidelines for intake of these as related to bone health are based on very little data.

### Summary and Conclusions

A summary of key messages focused on families is shown in Table 2. School age is a critical life phase for bone growth and development. This is especially true for a short window of several years beginning in early puberty. As such nutritional and health guidance related to bone health needs to focus on this age group. Although calcium is critical to provide the principal bone mineral, attention needs to also be paid to magnesium and phosphorus as well as vitamin D. Particular attention should be given to assessing bone mineral status of children with chronic illnesses and those with restrictive diets. There is a need for a global approach to understanding dietary bone health requirements, as most current data and recommendations are focused on Caucasian populations and North America and Europe.

**Table 2:** Key messages for families regarding bone mineral nutrition in school age children (1, 2).

• Calcium intake should include about 3 servings of dairy or calcium-fortified dairy substitutes such as soymilk beverages or calcium fortified juices and cereals.
• Adequate vitamin D intake requires multiple daily servings of milk or vitamin D fortified foods or consideration of a supplement, especially in low exposure situations.
• If uncertain as to the calcium or vitamin D content of a food, check the food label to identify calcium and vitamin D content.
• Limit soda intake to at most one serving daily if it cannot be completely excluded.
• Recurrent or unexpected fractures should be evaluated for possible bone loss disorders including biochemical evaluation and possible bone mineral content measurement using DXA with age-appropriate standards.
• Children with chronic illnesses should be assessed usually via DXA for possible bone demineralization using age appropriate standards especially if receiving a medication known to be associated with bone loss.

## Author Contributions

The author confirms being the sole contributor of this work and has approved it for publication.

## Conflict of Interest

The author declares that the research was conducted in the absence of any commercial or financial relationships that could be construed as a potential conflict of interest.

## Publisher's Note

All claims expressed in this article are solely those of the authors and do not necessarily represent those of their affiliated organizations, or those of the publisher, the editors and the reviewers. Any product that may be evaluated in this article, or claim that may be made by its manufacturer, is not guaranteed or endorsed by the publisher.
